# Psychological aspects of wound management following self-harm on psychiatric wards

**DOI:** 10.1192/bjb.2019.10

**Published:** 2019-04

**Authors:** Alla Rubitel

**Affiliations:** Consultant Psychiatrist in Psychotherapy, Central and North West London NHS Foundation Trust; email: alla.rubitel@nhs.net

I note with interest the initiative by Buick *et al* to evaluate a peer-led workshop on suturing skills for doctors working in psychiatric hospitals.^1^

The authors describe improved confidence of doctors with regards to suturing, associated with a reduction in transfers to accident and emergency (A&E) departments and a significant potential cost saving (calculated at £183.76 for each transfer that is avoided). It would be interesting to learn whether the intervention was associated with any change in the frequency of self-harm on the psychiatric wards.

Albert *et al* make important comments on the benefits for doctors in continuing to apply their basic medical skills in this context, and point to a cognitive–analytic model to describe role reciprocity in the case of self-harming patients having interventions for their wounds. It would be interesting to investigate further how in-house physical treatment may differ from A&E treatment concerning rescuer-to-rescued roles and reinforcement.

I would like to suggest that the decision to provide physical treatments (including suturing) – or the decision not to do so – will have an effect on the overall treating relationship and treatment frame.

What does it mean for a patient who expresses distress or hostility through cutting, when the doctor tasked with treating their psychological difficulties also becomes involved in the physically intimate act of suturing their body? How are the doctor's own emotional responses towards the patient's self-harm and the subsequent restorative procedure registered and managed when the doctor either performs the procedure directly, or sends the patient to A&E?

Perhaps these complex emotional factors could also be worked out in peer groups – in particular through Balint or other psychological case-based discussion groups.
